# The Speculum Uteri

**Published:** 1853-03

**Authors:** Henry Miller

**Affiliations:** Professor of Obstetric Medicine in the University of Louisville


*The Speculum Uteri.* By Henry Miller, M. D., Professor of Obstetric Medicine in the University of Louisville.

(From the Transactions of the Kentucky State Medical Society.)

*The Speculum Uteri.* The speculum uteri is not an instrument of modern invention, although it had fallen into disuse for ages, until it was revived by M. Recamier, in France, early in the present century. The speculum of Racamier was a concial tin tube, polished upon its internal and external surfaces, which he employed at first, of small size, for the purpose of making various applications, of a mild nature, to ulcerations of the vagina and os uteri, but subsequently of larger size, the more completely to expose the affected parts in cancer of the womb, and to apply caustics for its cure. The introduction of a tube, of such diameter as is required in some cases, being more or less difficult and painful, other practitioners had the speculum constructed of two or more branches or valves, which, when closed, form a concial tube that may be opened in the vagina, to any desired extent, by simple mechanical contrivances attached to the free extremity of the instrument.

Instead of describing the different specula which have been invented, and discussing their advantages and defects, the writer will be content to declare his own preference of the quadrivalved speculum for diagnostic, and not unfrequently, for therapeutic purposes. It may, when there is occasion, be easily converted into a trivalve or bivalve speculum, by detaching one or both of the articulating branches. The quadrivalve, which he uses, was made by Charriere, of Paris, and when fully expanded, its uterine extremity has an aperture of 2¼ by 2¾ inches.

The quadrivalve or bivalve speculum answers very well for cauterization, when the nitrate of silver, in stick or solution, is used; but if it be necessary to apply more powerful caustics, such as the potassa fusa or potassa cum calce, whose diffusion might do injury to the vagina, the tubular speculum should be employed. The tube is also preferable for applying leeches to the uterus, as they are liable to take refuge between the branches of the valvular speculum or fix themselves upon the vagina instead of the os uteri. It is necessary to have tubes of different sizes: the writer finds that three are sufficient for all practical purposes. His are made of silver, polished upon both surfaces, and are conoidal, being circular at their uterine extremity and oval at the free extremity—the circular aperture measuring 1¼ inches in No. 1; 1½ inches in No. 2; and 1¾ inches in No. 3. The extremities offer slopes, which incline toward each other and which are directed toward the pubes, in the introduction of the instrument. These specula are furnished with wooden stoppers, which project about an inch at the uterine end, in the form of the glans penis, to facilitate their introduction

*The Speculum as a means of Diagnosis.*—In a paper, on the use of the speculum, read before the *Royal Medical and Chirurgical Society*, May 28th, 1850, Dr. Robert Lee makes the assertion that in the two great classes of organic diseases of the uterus—malignant and non-malignant—and in all the displacements of the uterus, he has derived little or no aid from the speculum, in their diagnosis and treatment. The writer confesses his unfeigned surprise when this assertion, by an author of Dr. Lee's standing in the obstetric department of the profession, first arrested his attention, in perusing the report of his paper in the London Lancet. In the discussion which ensued, none of the distinguished gentlemen present appear to have noticed it or animadverted upon it in such terms as it deserves. Let us, then, inquire whether the speculum is indeed superfluous, first, in organic diseases, and secondly, in displacements of the uterus. It will be conceded, we presume, that inflammation is an organic disease, and that it is, moreover, the architect of numerous other diseases of the same class. Now, Dr. Lee virtually affirms that the speculum is not needed to discover the existence of inflammation of the cervix uteri, and upon this we join issue with him, being willing to stake the fortune of the speculum on its trial by a jury of our peers.

If the speculum be discarded, we cannot discover inflammation in this, its favorite lurking place, except by the symptoms that accompany it, or by the touch, in the usual mode of examination. Will the symptoms reveal it? Their uncertainty and the dimness of the light they shed, are proverbial. There may be pain or a sense of heat in one of the iliac regions, together with backache and neuralgia of the musculo-cutaneous nerves of one or both thighs. There may be frequent and painful micturition or tenesmic irritation of the rectum. The menstrual function may be deranged, and there may be leucorrheal discharge. But any or all of these symptoms may be present, and yet inflammation may not exist, while there may be inflammation, and few or none of these symptoms be complained of. Of the truth of these remarks no practitioner can be ignorant, who is much conversant with the diseases of females, and is familiar with the use of the speculum. The writer well remembers the case of a lady, the mother of two children, who miscarried in her third pregnancy, and suffered severely with her head for more than a year afterwards. She complained of fulness of the head, with more or less pain continually, and occasionally with very acute pain. On the part of the uterine system there was no evidence of any thing amiss, except that she did not conceive again, and menstruation, though regular, was scanty, seldom lasting more than a day, and amounting to a mere show. There was not, at any time, leucorrheal discharge, nor did she complain of pelvic pains, and yet when examined with the speculum, chronic inflammation, with hypertrophy of the uterine neck, was discovered. This was cured by the usual treatment: menstruation returned to its healthy type, and the cephalic symptoms gradually abated.

Can the touch detect inflammation of the cervix? This question might be answered by another; could a blind surgeon detect cutaneous inflammation by the touch? The truth is (and every accoucheur well knows it), none of our senses is more deceptive than the touch, or more frequently leads to mistakes. The only discovery which can be made by it, in the matter under consideration, might be made as well by any other instrument as by the finger, viz: the existence of morbid sensibility in the cervix uteri. When the inflamed cervix is pressed upon by the finger, the patient usually winces, and so she would were it pressed upon by a stick. Morbid sensibility may, however, exist independently of inflammation, and cannot, therefore, be regarded as furnishing conclusive evidence in such an investigation.

Upon the whole, then, the practitioner who relies on the symptoms and touch only, for his diagnosis in these cases, can never know of a surety that inflammation exists: he may surmise it, but cannot positively have any greater certitude than could a blind occulist concerning the existence and nature of inflammation of the eyes.

Ulceration belongs also to the class of diseases, in which, according to Dr. Lee's assertion, little or no aid is to be derived from the speculum — howbeit he is incredulous as to the occurrence of this morbid state, in the female sexual organs, except to a very limited extent. He says explicitly that he has never seen ulceration of the os and cervix uteri, which was not of a specific character, especially scrofulous and cancerous. To fortify himself in this position seems to have been the main object of his paper; for could it be proved that ulceration is a rare disease in these parts, the speculum might the more readily be driven from the field. Dr. Lee's clique, who rallied around him in the debate, felt equally with himself the necessity of expunging ulceration from the list of female sexual maladies. To accomplish this, they were forced to maintain that ulceration necessarily involves a palpable loss of substance. It is readily admitted that, in this sense, ulceration is a rare form of disease of the os uteri; we are not sure, indeed, that we have ever once met with it, nor have we a right to look for deeply excavated ulcers in such a situation. The mucous membrane alone is commonly implicated, and this is here of such exceeding tenuity that it cannot be dissected from the subjacent tissue. The nearest approximation to a dissection, which can be made by the most skillful anatomist, is to lift it up, in delicate patches, upon the point of a sharp lancet. Supposing the membrane to be destroyed, in its whole thickness, by the ulcerated process, there would not, therefore, be palpable loss of substance or any thing like an ordinary ulcer upon the skin, or even upon the mucous membrane of the intestines. But there is, nevertheless, what fulfills the definition of ulceration, namely, a solution of continuity, in a soft part, accompanied by a purulent discharge, for it may be brought to light by the speculum, and when wiped with a sponge, a raw and often a bleeding surface is exposed. What matters it, if Dr. Lee and his partisans choose to call it "abrasion," "excoriation," or by any other name. Such a surface, produced by morbid action, were only the epithelium destroyed, is ulceration; for there is solution of continuity and there is purulent secretion.

Ulceration of the os uteri is usually accompanied by inflammation, and the symptoms to which it gives rise are nearly the same, only there is more constantly purulent leucorrhea. But this discharge does not always attend it; for the secretion may be so slight as to be absorbed, and there may be purulent discharge without ulceration. Ulceration cannot, therefore, be predicated of any case from the symptoms only. It may be discovered by the touch, when the roughness of the affected surface is well marked, but in the very great majority of instances, nothing can be positively affirmed until the parts are brought under occular inspection. Of this, every day's experience convinces the writer more and more firmly. While inditing this report, he had occasion to examine a lady, from a distance, whom one of the most distinguished surgeons in this country, after examination by the touch alone, pronounced to be laboring under displacement of the womb, the organ being, as he assured her, perfectly free from disease: the writer was soon satisfied, by a specular, as well as tactual examination, that there was chronic ulceration of the os uteri, but no displacement of any kind!

The committee will next attempt to estimate the claims of the speculum, as a means of diagnosis, in displacement of the uterus, the other class of cases, in which Dr. Lee says it is of no value. None of these displacements is clearly indicated by the symptoms alone, except retroversio uteri occurring in the pregnant state, in which the sudden and total suppression of urine, together with the severe sufferings of the patient, points plainly enough to its existence. But in the non-gravid state, neither retroversion, nor anteversion, nor prolapsus (the most common of all the displacements) is accompanied by such symptoms as throw any satisfactory light on the subject. To the touch, at least, an appeal must be made, and through it we may learn that the organ is displaced, and the manner of its displacement; but we cannot learn its pathological condition, a capital hiatus in the information we are in quest of, for the speculum has taught us the frequent, nay, the almost constant co-existence of inflammation or ulceration of the cervix uteri. So true is this, that the writer can conscientiously declare that, since he has used the speculum freely in his practice, he has seldom seen an instance of prolapsus or retroversio uteri, uncomplicated with inflammation or ulceration of the cervix; and he is becoming more and more sceptical as to the existence of simple displacement of the uterus. His own view of the pathology of such cases is, that inflammation is the primary and essential disease, while the displacement is merely a sequence. Such is the doctrine advocated by Dr. James Henry Bennett, in his valuable practical work on "Inflammation of the Uterus," who attempts to explain the occurrence of prolapsus on the principle of the increased gravity of the uterus, acquired by inflammation. Dr. Meigs rejects the doctrine, and thinks he has most triumphantly refuted it by showing, as we think he has very conclusively, the insufficiency of the explanation. (*Females and their Diseases, p.* 137.)

But it does not seem to have occurred to Dr. Meigs that the doctrine may be true, while the explanation may be false. Grant the existence of inflammation of the cervix as the antecedent, and it may be that the irritation, established in the part and propagated to the neck of the bladder and to the rectum, will eventually cause prolapsus by the bearing-down efforts which it provokes, and this, we suspect, is the true etiology.

Be this as it may, and whether inflammation is the antecedent or the consequent of the prolapsus, the writer reaffirms, without the fear of successful contradiction, that inflammation or ulceration exists in nearly every case of displacement of the womb, and that it can be detected only by the speculum.

But Dr. Lee, as we have seen, not only renounces the speculum in the diagnosis, but also in the treatment of the whole class of diseases we have been considering. It is difficult to imagine the grounds of this renunciation. Can it be that the treatment of these diseases, by other means, has been so successful in his hands as to preclude the hope of improvement? If so, we sincerely congratulate him on his good fortune, in a field where all other practitioners, from time immemorial, have met with little else than discomfiture. For our own part, we are not ashamed to confess that, until we called the speculum to our aid, we were defeated on every hand or, at best, victory so seldom perched upon our standard, that we were bound to regard our success as fortuitous, rather than merited. We never cured a case of prolapsus by the pessary, or of long-standing leucorrhea, connected with inflammatory or ulcerative disease of the cervix, by constitutional treatment and the ordinary local appliances.

Such *fillibustering* may succeed in recent and trivial cases, but when the disease is more strongly intrenched, it can only be dislodged by a superior force operating directly and systematically upon it.

These uterine affections are essentially local in their nature: they owe their origin to local causes, and are most successfully treated by local remedies. But the remedies must be sufficiently potent to make an impression upon the disease. The sprinkling of an inflamed or ulcerated os uteri, with simple or medicated water, by means of a syringe (the only local remedies resorted to by the *fillibusters*,) cannot be more efficacious than such piddling ablutions upon other parts of the body. What would be thought of a surgeon who should attempt to cure an external chronic inflammation by squirting a little water or solution of lead or zinc upon it, two or three times a day?

The more potent remedies which are addressed to the affected part through the speculum are, chiefly, the local abstraction of blood by scarification or leeching, and superficial or deep cauterization, according to the circumstances of the case. It is not the design of the writer to enter into details on this part of the subject; he begs to refer the Society to practical works, particularly to Dr. Bennett's treatise, already alluded to. He will, nevertheless, submit a few annotations, suggested by his own experience in this branch of practice, which has been pretty extensive.

*First.* — Local depletion may be effected as well by scarification as by leeching, when the inflammatory congestion occupies the superficies of the os uteri, and ought to be preferred, because it may be done more expeditiously, and is far less revolting to the patient. When the inflammation is deep-seated, and there is little or no discoloration upon the surface, leeches should be employed, and half a dozen are commonly sufficient to procure as free bleeding as is desirable. Local blood letting is a valuable part of the treatment of these cases, and ought always to be premised, whenever there is any considerable degree of inflammation. It is a good preparation for cauterization, and may be advantageously repeated, in conjunction with cauterization, until the inflammatory congestion is subdued.

*Secondly.*—With the same view, cold mucilaginous injections—infusion of flaxseed or slippery elm—should be thrown into the vagina, by the patient, three times a day. But these will accomplish nothing unless a good syringe is provided, and the patient properly instructed in its use. The injections should be taken in a recumbent posture; the syringe ought to hold several ounces and have a pipe, with a bulbous end, long enough to reach the superior portion of the vagina.

*Thirdly.*—When the inflammation or ulceration is confined to the mucous membrane, with only slight enlargement and no induration of the cervix, cauterization with the nitrate of silver in substance, is the only application which will be found necessary in most cases. This ought not to be repeated too frequently—an error, which the writer has reason to believe, is committed by some,—not oftener than once a week. Six or eight of these hebdomidal cauterizations may suffice to cure the disease; but in some cases, a longer perseverance may be necessary, and in a few, the inflammation may prove altogether refractory. In such instances, the writer's practice is to cauterize once superficially with the potassa cum calce, and afterwards, with nitrate of silver as at first.

*Fourthly.*—Should the inflammation have extended to the proper tissue of the cervix, and resulted in induration, deep cauterization with the potassa cum calce will be indispensable to restore the part to its normal state, and heal any ulceration which may exist. It is quite useless to treat such a condition with the nitrate of silver: the ulceration will seldom be cured by it, and it can make no impression upon the deeper seated disease. The writer has practiced deep cauterization, in many cases; in several, he has used the actual cautery, and he has never known any serious accidents to follow. He is always careful, however, to apply the caustic through a tubular speculum, and to sponge off the part, so as to guard against any of the caustic remaining and spreading to the sound parts, after the withdrawal of the speculum. With this precaution, he considers it to be as safe to apply caustic to the cervix uteri as to the skin. Much obloquy has been cast upon the speculum, on account of alleged abuses of cauterization, and the writer doubts not that there is some foundation for it; for he can easily conceive that the careless or inexpert use of such a potent agent, may produce extensive inflammation and sloughing, followed by unnatural adhesions and contraction of the genital passage. But such consequences are attributable to the awkwardness or ignorance of the operator, and are no more chargeable to the speculum than is the transfixion of the vein in phlebotomy to the lancet. The writer can truly say that no such consequences have ever happened to him or need happen to any one, fit to be trusted with the speculum.

*Fifthly.*—Rest in a recumbent posture, more or less strictly guarded, according to the degree of inflammatory action that exists, is a material adjuvant in the treatment of these cases: and where this can not be enforced, the disease is greatly prolonged, and may prove altogether ungovernable.

Exercise or even the erect or semi-erect position tends, in a direct manner, to increase the uterine congestion and aggravate the sufferings of the patient. The writer cannot doubt, from what he has seen, that much mischief is often done by urging the patient to take exercise, under the fallacious idea that weakness is the sum total of her ailments, and that if she can only be strengthened by air and exercise, all will be well with her.

So strongly is the imagination of some physicians haunted with the bugbear, weakness, that they will persist in keeping the patient in motion, notwithstanding that every step is a dagger to her. When shall more rational views obtain currency in the profession? How long shall a mere effect engross the attention, while the cause is overlooked?

The writer was recently consulted in the case of a lady, who suffered greatly from pelvic pains after her second confinement, increased by exercise or the erect position. She had hemorrhagic discharges from the uterus for several weeks after parturition, with almost daily febrile excitement, intense thirst, loss of appetite, and general debility. The debility, unfortunately, absorbed the attention of her medical attendant, and to remedy this, exercise in a carriage was commenced on the eleventh day after her accouchment, and presisted in daily, in spite of her remonstrances, extorted by the increase of her suffering, and finally, she was sent away on an excursion in pursuit of the *ignis fatuus*, " strength. " When she returned home, a specular examination was made, and a high degree of inflammatory engorgement of the uterine neck and upper portion of the vagina, with ulceration around the os, was discovered, which had existed doubtless since her delivery.

*Sixthly*—Although the local treatment is paramount to every thing else, the state of the general system must not be overlooked or neglected. If constitutional irritation exist it must be subdued by appropriate remedies, or if any of the functions are sympathetically deranged, they must be restored to a healthy condition by suitable treatment. In recent cases, some degree of febrile excitement not unfrequently exists, and to allay this, it may be proper to put the patient upon an abstemious regimen, to purge actively every day or every other day, and if there be hardness as well as acceleration of the pulse, general blood-letting may be necessary.

Dr. Dewees was well aware, though he had not the ocular proof, of the existence of uterine and vaginal inflammation, in many instances of leucorrhea, which is only another name for the disease we have been considering, and the success of his treatment was doubtless attributable to the bleeding and purging he prescribed, rather than to cantharides, which he regarded as a kind of specific. This is fairly to be inferred, from the fact that none of his cotemporaries or successors have been as fortunate in the use of cantharides as himself, which can be accounted for only by supposing that they have relied principally upon the specific, to which the multitude are always prone, to the neglect of due attention to the state of the system. It is not intended to be asserted that cantharides is devoid of all remedial virtues in these cases. By its action upon a contiguous and associated viscus, it may exert some beneficial influence upon the genital organs; nevertheless we are persuaded that the antiphlogistics, so vigorously employed by Dr. Dewees, had a larger share in extinguishing the disease than had the cantharides, pushed ever so often *usque ad stranguriam*.

In more protracted cases, the general state is characterized by veritable debility, a languid circulation, coldness of the extremities and impaired digestion and assimilation.

Under such circumstances, it will be proper to administer tonics, especially some of the preparations of iron, and to regulate the secretions and excretions by the use of alteratives and purgatives The selection of these will be governed by the indications of each particular case. As to purgatives it is necessary to observe that only such of them are admissible as may be required to procure one full alvine evacuation daily, to effect which, a pill or two of rhubarb and extract of colocynth, or of rhubarb, aloes and soap, may be taken every night.

Mercury, iodine, arsenic and antimony are among the most powerful alteratives, and the indications for the use of remedies of this class may be fulfilled by the various preparations and combinations of these agents.

As to sarsaparilla, which is so often prescribed, we do not know that we have ever obtained any good from it, even when furnished by the regular apothecary; while sure we are, that the quackish preparations of it, which find their way by the hogshead into the stomachs of our nostrum-loving population, are utterly worthless.

